# Supplementary material for: FAST4D—A New Score to Reduce Missed Strokes in Emergency Medical Service: A Prospective, Multicentric Observational Proof-of-Concept Trial
Source: J Clin Med. 2024 Aug 25;13(17):5033. doi: 10.3390/jcm13175033 (PMC11396033; doi:10.3390/jcm13175033)
Supplement: Supplementary file 1 [file jcm-13-05033-s001.zip › jcm-3139744-supplementary.pdf]

# Supplement

## Baseline data of the whole cohort

| | AIS | ICH | total |
|---|---|---|---|
| Total (n) | 926 | 56 | 995 |
| Age (mean ± SD) | 74 ± 14 | 75 ± 11 | 74 ± 14 |
| Female Sex (n) | 471 | 32 | 503 |
| Diagnosis by MRI (n) | 750 | 11 | 761 |
| Diagnosis by CT (n) | 213 | 61 | 274 |
| IVT (n) | 141 | 0 | 141 |
| EVT (n) | 63 | 0 | 63 |

*Table S1: Baselinecharacteristics of the included patients. AIS = acute ischämic stroke, ICH = intracerebral hemorrhage, EMS = emergency medical service, IVT = intravenous thrombolysis, EVT = endovascular thrombectomy, MRI*

## Detection of Posterior Circulation Strokes

After directly comparing both tests in terms of the incidence of posterior circulation strokes, we will proceed to analyze each test in greater depth with respect to this specific type of stroke. Out of the 1469 patients, 314 were diagnosed with a posterior circulation stroke. Among these, at least one of the items of the new FAST4D score was positive in 298 patients, while 16 patients did not exhibit any positive FAST4D score items. The sensitivity of the novel FAST4D score in detecting posterior circulation strokes was 95,0% ($\chi^2 = 0.14$; $p = 0.71$; see Table S2).

| | | FAST4D positive | | |
|---|---|---|---|---|
| | | no | yes | total |
| **Posterior circulation stroke** | No | 53 | 1102 | 1155 |
| | yes | 16 | 298 | 314 |
| | total | 69 | 1400 | 1469 |

*Table S2: 4-field panel of the novel FAST4D score compared with the diagnosis of a posterior circulation stroke.*

When compared to the traditional FAST score alone, among 314 patients diagnosed with a posterior circulation stroke, at least one of the items of the FAST score alone was positive in 175, whereas in 139 patients with a posterior circulation stroke, none of the traditional FAST items was positive. The sensitivity of the traditional FAST score in detecting strokes in the posterior circulation was (55,7%, $\chi^2 = 34.2$; $p < 0.001$; see Table S3).

| | | FAST positive | | |
|---|---|---|---|---|
| | | no | yes | total |
| **Posterior circulation stroke** | No | 313 | 842 | 1155 |
| | yes | 139 | 175 | 314 |
| | total | 452 | 1017 | 1469 |

*Table S3: 4-field panel of the traditional FAST score compared with the diagnosis of a posterior circulation stroke.*

## Overtriage

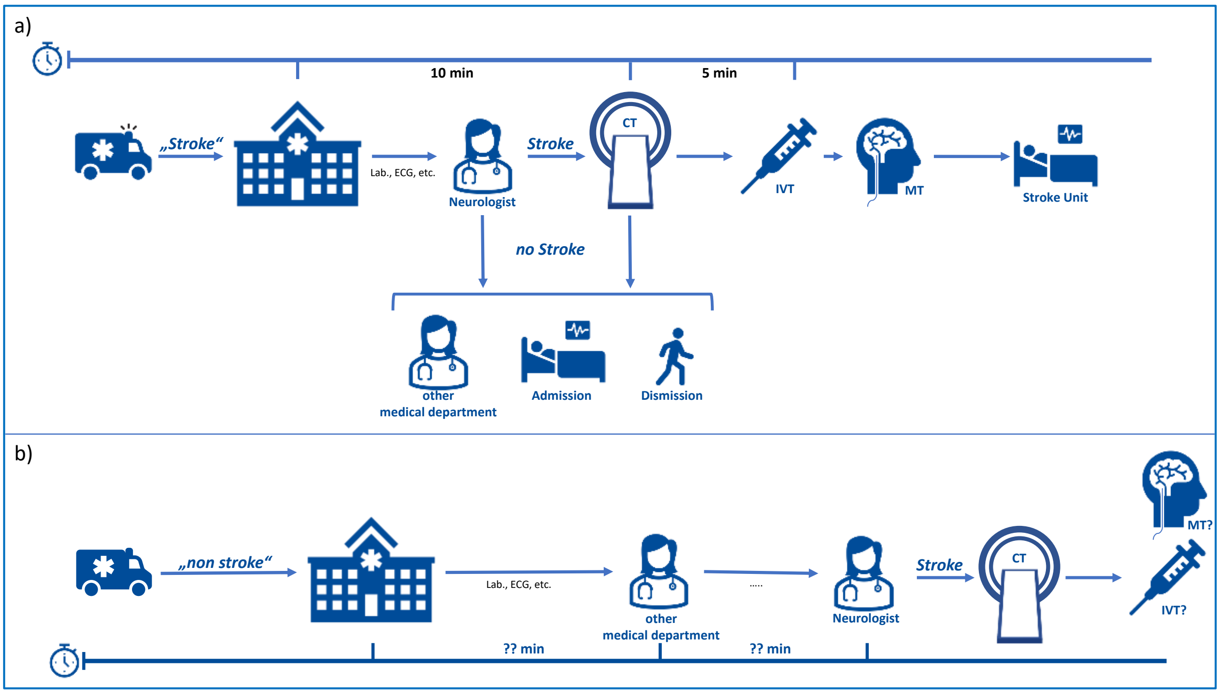


*Figure S1: The figure shows the emergency care procedures for **a)** patients with suspected and **b)** patients with another suspected diagnosis. An increase in the detection rate leads to an increased rate of patients incorrectly recognized as stroke. In emergency medicine, such cases are called overtriage. An overtriage must always be weighed between benefits for the correctly recognized patient and damage caused by the incorrect assignment. Furthermore, an overtriage binds personnel and technical capacities. **a)** In order to recognize as many patients with stroke as possible, overtriage is inevitable to ensure the fastest way to recanalization therapy. **b)** Stroke patients who did not reach the Emergency department as stroke will have a prolonged approaches until the diagnose stroke occurs and therefore a higher cerebral damage.*

The majority (76%) of stroke patients were correctly identified as stroke cases by the emergency medical service using the FAST4D score. The "overtriage" rate was only 25%, revealing few non-neurological conditions alongside typical neurological "stroke mimics", all of which could be safely managed in the emergency department as you can find in table SS4.

| MEDICAL DISCIPLINE | TOTAL | PROZENT |
|---|---|---|
| NEUROLOGY | 303 | 70% |

| | | |
|---|---|---|
| INTERNAL MEDICINE | 75 | 17% |
| PSYCHIATRY | 32 | 7% |
| OTHERS | 24 | 6% |
| **TOTAL** | **434** | **100%** |

*Table S4: Overview of medical conditions to their corresponding medical specialty in patients mistakenly assigned as stroke cases (overtriage).*
